# Synergism between nonane and emanations from soil as cues in oviposition‐site selection of natural populations of *Anopheles gambiae* and *Culex quinquefasciatus*

**DOI:** 10.1186/s12936-020-03575-0

**Published:** 2021-01-21

**Authors:** Victor S. Mwingira, Leonard E. G. Mboera, Willem Takken

**Affiliations:** 1 Laboratory of Entomology, Wageningen University & Research, P.O. Box 16, 6700 AA Wageningen, The Netherlands; 2 SACIDS Foundation for One Health, Sokoine University of Agriculture, Chuo Kikuu, P.O. Box 3297, Morogoro, Tanzania

**Keywords:** Oviposition, Pheromone, Nonane, Breeding-site soil, *Anopheles gambiae*, *Culex quinquefasciatus*

## Abstract

**Background:**

Olfactory cues have been shown to have an important role in guiding gravid mosquito females to selected sites for egg laying. The objective of this study was to determine the influence of emanations from soil from a breeding site and the putative oviposition pheromone nonane on oviposition-site selection of natural populations of *Anopheles gambiae sensu lato* (s.l.) and *Culex quinquefasciatus*.

**Methods:**

This field-based study was conducted in Mvomero District in East-central Tanzania. In a dual-choice experimental set up, clay bowls were dug into the ground and filled with one of the following treatments: (i) distilled water + autoclaved soil (control), (ii) distilled water + soil from a natural mosquito breeding site, (iii) distilled water + nonane and (iv) distilled water + nonane + soil from a natural breeding site. Soil was dried and autoclaved or dried only before use. After five days of incubation, larvae were collected daily for 10 days. The median number of larvae per bowl per day was used as outcome measure.

**Results:**

Autoclaved soil had a significant attractive effect on oviposition behaviour of *Cx. quinquefasciatus* (median values ± s.e: 8.0 ± 1.1; *P* < 0.005) but no effect on *An. gambiae* (median value ± s.e: 0.0 ± 0.2; *P* = 0.18). Nonane and emanations from untreated soil significantly and positively influenced the selection of oviposition sites by both *An. gambiae* s.l. (median values ± s.e.: 12.0 ± 2.0 and 4.5 ± 1.5, respectively; *P* < 0.0001) and *Cx. quinquefasciatus* (median values ± s.e.: 19.0 ± 1.3 and 17.0 ± 2.0, respectively; *P <* 0.0001). A mixture of nonane and untreated soil caused a synergistic effect on oviposition behaviour in *An. gambiae* s.l. (median value ± s.e.: 23.5 ± 2.5; *P* < 0.0001) compared to either nonane (median values ± s.e.: 12.0 ± 2.0; *P* < 0.0001) or untreated soil alone (median value ± s.e.: 4.5 ± 1.5; *P* < 0.0001). A synergistic effect of nonane mixed with untreated soil was also found in *Cx. quinquefasciatus* (median value ± s.e.: 41.0 ± 2.1; *P* < 0.0001) compared to either nonane (median value ± s.e. 19.0 ± 1.3; *P < 0.0001*) or untreated soil alone (median value ± s.e.: 17.0 ± 2.0; *P* < 0.0001). The oviposition activity index for *An. gambiae* was 0.56 (*P* < 0.001) and for *Cx. quinquefasciatus* 0.59 (*P* < 0.0001).

**Conclusions:**

The larval pheromone nonane and emanations from breeding-site soil both induced oviposition in wild *An. gambiae* s.l. and *Cx. quinquefasciatus*, with a synergistic effect when both stimuli were present simultaneously. This is the first study in which nonane is shown to cause oviposition under natural conditions, suggesting that this compound can potentially be exploited for the management of mosquito vectors.

## Background

Vector control is a fundamental element of the existing global strategy to fight mosquito-borne diseases [1]. Existing mosquito control programmes have been developed based on understanding of the behaviour and lifecycle of the vectors. The use of insecticide-treated nets (ITNs) and the recently developed toxic sugar bait technique resulted from the exploitation of blood and plant host-seeking behaviour of mosquitoes, respectively [2–5]. Additionally, indoor residual spraying with insecticides (IRS) is based on the observation that mosquitoes rest on walls after feeding and thereby come into contact with insecticides [6]. Larviciding and other environmentally-based interventions [7], on the other hand, are based on observations that mosquito eggs are laid on water and immature stages develop in water. Oviposition has been considered to be an important target to exploit for the control of mosquito-borne diseases [8–10].

Currently, the main effective methods of mosquito-borne disease control are the use of ITNs and IRS [11]. However, these methods are challenged by the wide-spread development of insecticide resistance [12–14] and the observed behavioural adaptation of mosquitoes to avoid insecticides [15]. Mosquitoes can detect the presence of insecticides from treated surfaces and divert their biting activities in time and space to exploit untreated resources [16–18].

In recent years, a change in host-seeking behaviour of malaria mosquitoes was reported in response to the Africa-wide coverage of ITNs: considerable shifts in biting time [19–22] and place, i.e. outdoors vs. indoors [23, 24] were found in both East and West Africa. Moreover, in certain areas malaria vectors have shifted their blood-host preference to other vertebrate species as a result of insecticide use [25]. In such circumstances, the core interventions of ITNs and IRS need to be supplemented by larval source management, which includes vector habitat modification, habitat manipulation, larviciding and biological control [11].

One of the possible options is habitat manipulation by using infochemicals derived from mosquito breeding sites to lure vectors into death traps [26–28]. Already habitat-derived infochemicals have been used to enhance the trapping of gravid *Culex quinquefasciatus* [29–31] and *Aedes aegypti* [32, 33] mosquitoes in autocidal oviposition traps. The use of infochemicals from breeding sites to lure gravid mosquitoes has the potential to target egg-laying adults [26, 34]. Thus, infochemicals that direct gravid mosquitoes to lay eggs in selected habitats are likely to be the focus of future vector control strategies.

These strategies are relevant because most insects express a preference for oviposition habitats that improve survival, growth and reproductive potential of their offspring, especially for species in which juveniles are incapable of migrating away from poor-quality habitats [35]. Sites selected for oviposition by mosquitoes can vary from few to many, often within a few days, and depending on topography and rainfall. Especially when they are few, can easily be targeted for control measures. Oviposition-habitat selection is particularly relevant in insect vectors of medical importance as it determines the localities to which larvicidal control measures need to be targeted [36].

The search for oviposition attractants is aimed at discovering a chemical compound or blends of compounds that attract selected species [37]. Skatole and (5R,6S)-6-acetoxy-5-hexadecanolide were discovered to be oviposition cues for *Cx. quinquefasciatus*, and a blend of these compounds caused a synergistic response in gravid mosquitoes [38]. Recently, it was reported that gravid mosquitoes of *Anopheles gambiae* are attracted to cedrol, a compound identified from a natural breeding site, as oviposition cue [39]. Cedrol was found to be derived from grass species found in breeding sites of *An. gambiae sensu stricto* (s.s.) [28]. Additionally, nonane, a compound identified in the headspace from mosquito larval habitats in the laboratory, was found to be attractive to gravid *An. gambiae* [40]. Nonane is a volatile chemical compound with nine carbon atoms. A related chemical compound with nine carbon atoms similar to nonane and which acts as an attractant to mosquitoes is nonanol, which is known to attract *Cx. quinquefaciatus* [41]. Current evidence suggests that species-specific as well as habitat-derived chemicals affect oviposition behaviour of mosquitoes.

The objective of the present study was to explore the influence of habitat-derived infochemicals and nonane on the selection of oviposition sites by *An. gambiae* and *Cx. quinquefasciatus* under field conditions. Studies were done (i) to establish the most effective (artificial) oviposition device for field use, (ii) to examine the effect of soil-derived infochemicals and of the putative oviposition cue nonane and (iii) to investigate the interaction between emanations from breeding-site soil [42] and nonane on oviposition behaviour of wild *An. gambiae* and *Cx. quinquefasciatus*.

## Methods

### Study area

The study was carried out in Mvomero District in east-central Tanzania (5° 47′ 09″–7° 23’40’’ S, 37°11′ 09″–38°01′ 33″ E), between March and June 2012. This area has typical tropical characteristics: temperatures oscillating between 19 °C and 31 °C, RH > 80%, and annual rainfall of 1146 mm (based on data collected from Mtibwa meteorological station, 2008–2013). The area has a bi-modal type of rainfall with with long rainy season from March to June and a short one from October to December, with a relatively short dry spell between July and September.

Digoma village was selected for the field experiments; the village borders the Nguu mountains and receives water from rivers which flood the valleys. This enables irrigated rice production in the river basin throughout the year. In addition to rice production, therefore, the area has favourable environmental conditions for mosquito production. Malaria and lymphatic filariasis are the most common mosquito-borne diseases in the area [43]. The most abundant mosquito vectors in the area include *An. gambiae* s.s., *Anopheles arabiensis*, *Anopheles funestus* and *Cx. quinquefasciatus*. *Anopheles gambiae* s.s. and *An. arabiensis* are genetically related and morphologically indistinguishable, and are here grouped as *An. gambiae* unless otherwise mentioned.

### Oviposition containers

Containers used for oviposition in this experiment included clay pots, plastic bowls, aluminium pans and plates which were either blue or transparent in colour. With the exception of aluminium plates, which had a diameter of 27 cm and a depth of 4 cm, all other containers were of similar size (average diameter of 25 cm and a depth of 7 cm).

### Distilled water

Distilled water was used in the experiment to dilute chemicals and obtain the desired dosages, and also to dissolve oviposition substrates before setting up the experiments. Distilled water was also used for rinsing all washable items used in the experiments. It was produced and packed by LAL Laboratories, Tanga, Tanzania. Distilled water was used alone in the early experiments as oviposition substrate and in the control arm. For experiments involving nonane, a control solution was used, which consisted of 55% v/v distilled + 40% v/v methanol + 5% v/v tween20. Previous studies had not found any behavioural or larvicidal effect of this mixture [40].

### Soil

Clay soil originated from a natural breeding site in Mvomero that contained early-stage larvae of *An. gambiae*. After collection, soil was air dried before further use. Two hundred gram of dry soil was added to each container to simulate natural conditions of breeding sites. Previous studies have shown that volatile emissions associated with microbial organisms in the soil mediate the location of potential mosquito habitats [44]. Therefore, a fraction of the dried soil samples was autoclaved twice for 15 min. at 130 °C and 1.4 kg/cm^2^ pressure and allowed to cool down to kill any organisms that might be involved in the production of volatile chemicals [44, 45]. When the containers were filled with the treatment solution, the maximum depth was 8 cm.

### Chemical cues

Nonane (Lot and filling code: 132995235107188, ≥ 99.0%; Sigma Aldrich Chemie BV, Zwijndrecht, The Netherlands) was selected as chemical cue to lure gravid mosquitoes (Schoelitsz et al. [40]). Nonane is insoluble in water and, therefore, it was dissolved in methanol and tween20 in the following ratio: 55% v/v of nonane + 40% v/v methanol + 5% v/v tween20. The mixture was further diluted with distilled water to achieve a nonane concentration of 5.5 × 10^− 5^M. In experiments with nonane, the compound was tested at a concentration of 5.5 × 10^− 5 ^M and it was paired with a control solution of distilled water + methanol + tween20 (see above).

### Selection of artificial oviposition containers

To simulate natural breeding sites, experiments were conducted to search for the most preferred artificial breeding site for a natural population of mosquitoes. A range of man-made liquid containers was evaluated in the field in order to identify the most suitable oviposition container for mosquitoes in the area. These included plastic bowls (blue and transparent), aluminium plates and pans, and clay pots. To explore possible colonization of artificial habitats by wild mosquitoes, 25 containers were placed randomly in an open sunlit field. Five lines, separated by 3 m, each composed of five containers that were placed at 3 m distance from each other in the ground, with the top of the container being at ground surface level. Containers were filled with distilled water to capacity and were checked daily for the presence of larvae/pupae for a period of 10 days. Evaporated water was replenished with an equal amount of water in each container daily. Distilled water had already been successfully used as oviposition substrate for mosquitoes in the laboratory and semi-field environment [40]. Therefore, the aim of this experiment was to test if a natural population of mosquitoes would oviposit in these simulated breeding sites containing distilled water. The container that produced the highest number of larvae was selected for the behavioural experiments.

### Site selection for oviposition trial

Four sites (north, south, east and west) were selected for the dual-choice oviposition trial in an area covering a total of 4 hectares on both sides of a river near Digoma village, N.E. Tanzania. This area was chosen based on the following criteria: proximity to the river basin, presence of rice fields, absence of flooding, open to sun and proximity to human settlements. Rice growing is the main economic activity. All sites were surrounded by a wire mesh to prevent humans, animals or frogs from interfering with the experiments. In addition, a local field worker was hired to oversee the site during the entire study period.

### Clay pots

**Fig. 1:**
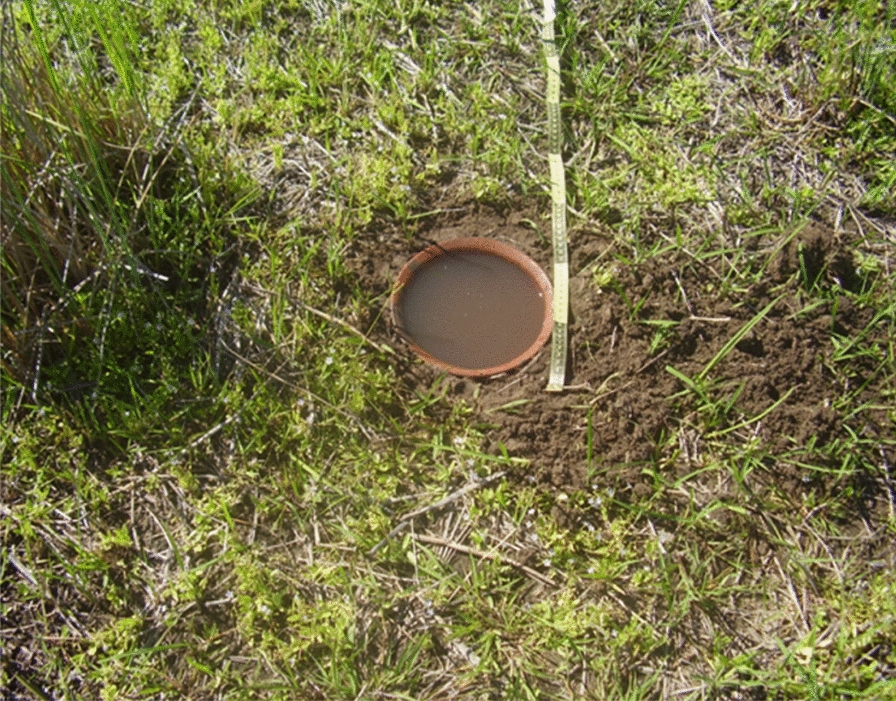
A clay pot placed into the field with the edge level with the surrounding ground level. These pots were used as proxy for natural oviposition sites (Photograph: VSM)

As clay pots gave the best result as oviposition container (see above), they were selected for the remainder of the study. The pots had an average diameter of 200 mm and a depth of 100 mm was used as artificial breeding sites for the field trial. They were made locally from clay soil, moulded by hand to make a bowl-shaped pot and left to dry, where after they were cured by fire. Clay pots were positioned in the ground so that the margins of the pots were level with the surrounding ground (Fig. 1). The pots were placed in the valley plain, within a rice field in the vicinity of a village.

### Design of oviposition experiments

Clay pots were placed at selected sites in the field 72 h before the start of the experiment and filled to capacity with distilled water; water was replenished until the clay reached saturation. Prior to the start of the experiment, the clay pots were emptied and immediately filled with 1L of the oviposition substrate (treatment or control) one h before sunset. Oviposition pots were left undisturbed for five days and from the 6th day, pots were inspected every morning and larvae were collected and recorded daily from 06:30 am for the next 10 days. Whenever the water level decreased in the pots, distilled water was added to maintain the water level. Collected larvae were then transferred to a temporarily established local laboratory together with the water from the pot and reared under controlled conditions. This water was used as rearing substrate in the laboratory for the first 24 h. After that time, larvae from each oviposition pot were transferred to mosquito rearing bowls, which contained distilled water. Rearing bowls were placed below light bulbs and larvae were fed Tetramin® fish food twice daily. Larval growth and development were observed and recorded until pupation and adult formation. Oviposition pots containing experimental substrates remained in the field for 15 d after which the substrates were removed; the pots were cleaned and replaced. For each pair of treatment and control, the pots were oriented facing East and West positions, and these positions were switched for each replicate.

### Dual‐choice tests

The effect of substrate on oviposition choice of wild mosquitoes was tested in a dual choice test, where one clay pot contained the treatment substrate and the other pot the control substrate (Table 1). Treatment and control were placed 3 m from each other. Each treatment pair was replicated 40 times, 10 pairs at four different sites (see site selection above); pots with substrate were incubated in the field for five days, and then examined for the presence of larvae for 10 days. Newly emerged larvae were collected daily. For each replicate, the positions of the treatment and control were switched each time to counterbalance the effects of wind direction.

**Table 1:** Experimental treatments for the dual-choice tests

Treatment series	Substrate A	Substrate B	No. of replicates
1	Distilled water + autoclaved soil	Distilled water	40
2	Distilled water + untreated soil	Distilled water + autoclaved soil	40
3	Distilled water + autoclaved soil + nonane	Distilled water + autoclaved soil	40
4	Distilled water + autoclaved soil + nonane	Distilled water + untreated soil	40
5	Distilled water + untreated soil + nonane	Distilled water + untreated soil	40

### Influence of autoclaved soil from a natural breeding site

A total of 200 g of autoclaved soil from a natural anopheline breeding site + distilled water in a clay pot and tested against distilled water only. Pots were each filled with 1250 ml distilled water.

### Influence of untreated soil from a natural breeding site

Clay pots were filled with 200 g of dried soil from a natural anopheline breeding site. To test whether soil produces chemical cues or acts only as a visual cue for gravid mosquitoes [26], the soil was tested against autoclaved soil. The pots were each filled with 1250 ml of distilled water.

### Influence of nonane

1250 ml of the nonane solution + autoclaved soil was tested against 1250 ml of distilled water + autoclaved soil. Pots were filled with either 200 g autoclaved soil and distilled water or 200 g autoclaved soil and a nonane solution.

### Influence of soil from a natural breeding site and nonane

To investigate the interactive effects of breeding-site soil and nonane, combinations of both candidate stimuli were tested alone or as a mixture: (a) nonane + breeding-site soil against nonane + autoclaved breeding soil and (b) nonane + breeding-site soil against distilled water + breeding-site soil. Pots were filled with 200 g of soil and distilled water or a nonane solution until capacity.

### Mosquito species composition

All larvae collected were transferred to the insectary and reared until adult emergence. Newly emerged anopheline adults were identified to species level using morphological keys [46]. *Anopheles gambiae* specimens were preserved in Eppendorf tubes, which contained silica gel for further identification to distinguish between sibling species in the *An. gambiae* complex. Genotypic identification was conducted by using the ribosomal DNA-polymerase chain reaction (PCR) to separate *An. gambiae* s.s. from *An. arabiensis* [47]. Culicine mosquitoes were identified as *Cx. quinquefasciatus* or other culicines.

### Data analysis

SPSS 14 for Windows® was used to conduct Wilcoxon signed-rank tests for paired samples in order to determine the difference in the number of larvae in each oviposition bowl as an indicator of number of eggs laid. A Friedman test for multiple samples was used to determine the oviposition preference among several containers. The preferences of mosquitoes for ovipositing on different treatments were evaluated based on container index (CI) (% bowls harbouring larva). All statistical tests were conducted by using absolute numbers of larvae in pots as a proxy for the number of eggs laid in the pot.

The larval density index (LDI) was defined as the total number of larvae found divided by the total number of oviposition containers with larvae.

The oviposition active index (OAI) was used to determine the attractiveness of the treated substrate compared to control. It was calculated according to the formula; OAI = Nt − Nc/Nt + Nc [48]. Where Nt = number of larvae on the test substrate and Nc = number of larvae on the control substrate. In this study, it was observed that anopheline eggs, which are black in colour, tend to stick to the surface of the clay pot, which is also black. This poses a challenge to accurately score the number of eggs as a measure of oviposition activity of gravid females. Therefore, the number of larvae was scored as a proxy for the eggs that were laid in respective pots.

## Results

### Mosquito species composition

During the study on oviposition site selection and containers (see below), a total of 1,349 anopheline larvae and culicine 2,815 larvae were collected. All anopheline larvae collected in the containers consisted of *An. gambiae*. Molecular analysis (by PCR) of a subsample of 200 *An. gambiae* larvae revealed that 86% were *An. gambiae* s.s. and 16% *An. arabiensis*. Culicine larvae were identified as *Cx. quinquefasciatus*.

### Mosquito oviposition‐site selection between different substrates and containers

**Fig. 2:**
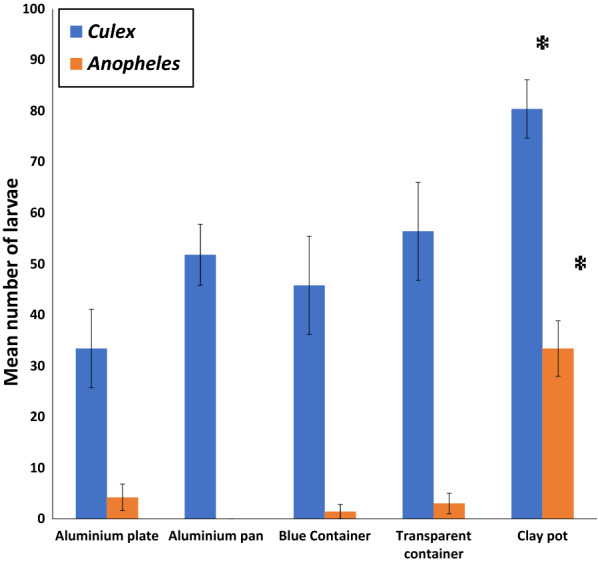
The mean number ± S.E. of larvae resulting from oviposition by a natural population of mosquitoes in a multiple choice set-up of oviposition containers. Asterisks indicate statistical differences from other treatments for a given species (*p < 0.05, Friedman test). The blue bars represent anophelines while the red bars represent culicines

Preliminary experiments showed that natural populations of *Cx. quinquefasciatus* oviposited in containers that were filled with distilled water. By contrast, a natural population of *An. gambiae* mosquitoes did not oviposit in containers filled with distilled water only. After adding 200 g of soil from a known anopheline breeding site to each container, significantly more larvae were found in clay pots than in other containers (Fig. 2). On average, 80.4 ± 5.7 (χ^2^ = 14.97, *p* = 0.005) larvae of *Cx. quinquefasciatus* and 33.4 ± 5.4 (χ^2^ = 9.92, *p* = 0.042) larvae of *An. gambiae* were found in clay pots containing soil. It was therefore decided to use clay pots for all successive experiments as proxies for natural breeding sites.

### Influence of chemical cues from soil

In a choice assay between distilled water with breeding-site soil and distilled water with autoclaved soil, the accumulated total number of *An. gambiae* larvae found in pots containing breeding-site soil left for 10 d was 254, with an average of 6.35 ± 1.16 larvae per pot per day. The number of *Cx. quinquefasciatus* larvae was 644, with an average of 16.1 ± 1.6 larvae per pot per day. The number of larvae found in pots containing breeding-site soil was significantly higher than that in pots with autoclaved soil for both *An. gambiae* and *Cx. quinquefaciatus* (z = − 4.016, P < 0.0001 and z = − 4.32, *P <* 0.0001 respectively). There were only few larvae of *An. gambiae* found in pots containing autoclaved breeding-site soil (Table 2).

**Table 2:** Mean number of larvae from a natural population of *Anopheles gambiae* s.l. that oviposited in clay pots filled with distilled water (DW), and distilled water + autoclaved soil (AC), distilled water + untreated soil (BS) and distilled water + nonane in a dual choice set-up in the field

Oviposition substrate	Pot positivity^a^		Quantity of larvae in pots		OAI	*p*-value
	No.	LDI	No. (%)	Median ± SE		
DW + AC soil	2	3.5	7 (1.7)	0.0 ± 0.2	1	0.18
DW	0	0	0 (0)	0		
DW + AC soil	2	5.5	11 (0)	0.0 ± 0.3	0.9	0.0001
DW + BS soil	22	11.6	254 (100)	4.5 ± 1.5		
DW + AC soil	3	5.3	16 (0)	0.4 ± 0.3	0.9	0.0001
DW + AC soil + nonane	32	15.7	503 (100)	12.0 ± 2.0		
DW + BS soil	14	23	322 (42.8)	8.1 ± 2.3	0.1	0.097
DW + AC soil + nonane	22	19.5	429 (57.2)	11.0 ± 2.1		
DW + BS soil	24	11.5	276 (21.7)	7.0 ± 1.3	0.6	0.0001
DW + BS soil + nonane	38	25.6	972 (78.3)	23.5 ± 2.5		

Larval density index (LDI), oviposition activity index (OAI) and *p*-values are shown

^a^No. of pots per treatment: n = 40

### Influence of nonane

In a choice assay between control + autoclaved soil + nonane and control + autoclaved soil only, a total of 503 larvae of *An. gambiae* were found in 32 out of 40 clay pots containing nonane, while a total of 825 larvae of *Cx. quinquefasciatus* were found in all 40 clay pots containing nonane. Larvae of *An. gambiae* were mainly found in pots containing nonane, with an average number of 12.6 ± 1.6 larvae per pot per day, whereas larvae of *Cx. quinquefasciatus* were found in both treated and control pots with an average of 20.6 ± 1.1 larvae per pot per day found in nonane pots and 5.7 ± 1.0 in control pots. The number of larvae found in pots containing nonane was significantly higher than the number of larvae found in the control pots for both *An. gambiae* and *Cx. quinquefasciatus* (z = − 4.978, *P <* 0.0001 and z = − 3.846, p < 0.0001, respectively) (Tables 2 and 3).

**Table 3:** Mean number of larvae from a natural population of *Culex quinquefasciatus* that oviposited in clay pots filled with distilled water (DW), and distilled water + autoclaved soil (AC), distilled water + untreated soil (BS) and distilled water + nonane in a dual choice set-up in the field

Oviposition substrate	Pot positivity^a^		Quantity of larvae in pots		OAI	*p*-value
	No.	LDI	No. (%)	Median ± SE		
DW + AC soil	24	10.5	252 (61.6)	8.0 ± 1.1	0.2	0.005
DW	20	7.9	157 (38.4)	2.0 ± 1.1		
DW + AC soil	22	9.6	210 (24.6)	5.5 ± 1.1	0.5	0.0001
DW + BS soil	32	20.1	644 (75.4)	17.0 ± 2.0		
DW + AC soil	20	14.9	229 (21.7)	3.0 ± 1.3	0.6	0.0001
DW + AC soil + nonane	40	20.6	825 (78.3)	19.0 ± 1.3		
DW + BS soil	36	14.4	517 (37.9)	13.5 ± 1.2	0.2	0.0001
DW + AC soil + nonane	40	21.2	847 (62.1)	20.0 ± 1.6		
DW + BS soil	34	12.2	414 (20.6)	11.0 ± 1.1	0.6	0.0001
DW + BS soil + nonane	40	39.9	1599 (79.4)	41.0 ± 2.2		

Larval density index (LDI), oviposition activity index (OAI) and *p*-values are shown

^a^No. of pots per treatment: n = 40

### The influence of nonane and soil from a breeding site

**Fig. 3:**
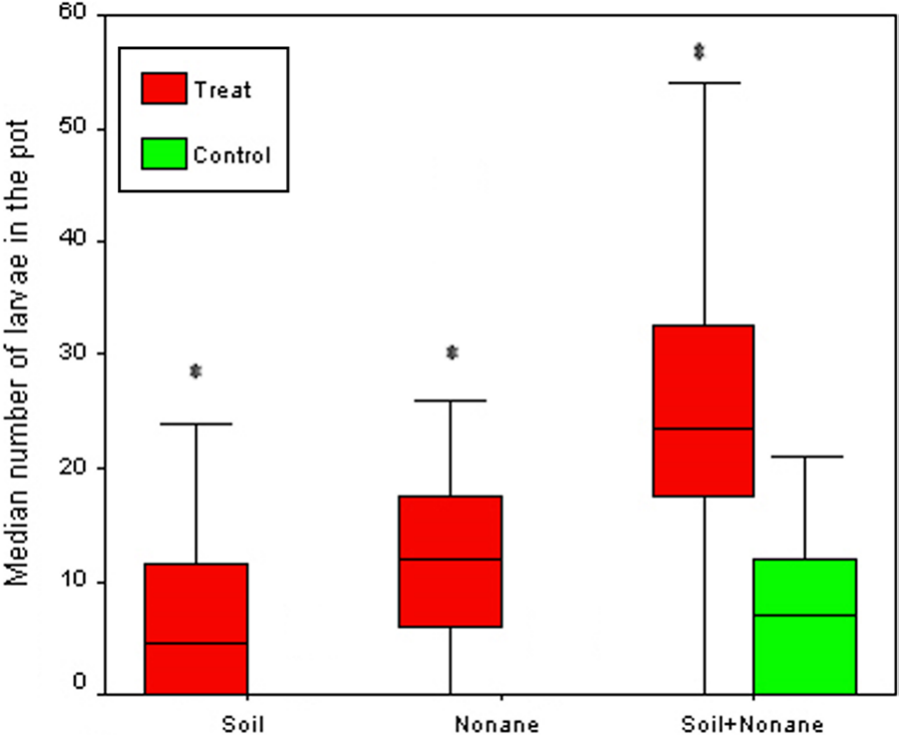
Oviposition response of *Anopheles gambiae* s.l. to a substrate containing soil, nonane or soil + nonane in a dual-choice field study. Distilled water + autoclaved soil was used as control. Boxplots represent the median number + quartiles of larvae per clay pot per day; Median and quartiles are given; asterisks indicate statistical differences between treatment and control for a given pair (*p < 0.05, Wilcoxon signed rank test)

In a choice test between distilled water with autoclaved soil + nonane against distilled water with breeding-site soil, a total of 751 *An. gambiae* larvae were found. Of these, 57.1% were found in pots containing autoclaved soil + nonane and 42.9% were found in pots containing breeding-site soil (Table 2; Fig. 3). Also, a total of 1,364 larvae of *Cx. quinquefasciatus* were found; 62.1% of these were found in pots containing autoclaved soil + nonane and 37.9% were found in pots containing breeding-site soil (Table 3). There was no significant difference between the number of larvae found in pots containing autoclaved soil + nonane and pots with breeding-site soil for *An. gambiae* (z = − 1.658, *P <* 0.097). However, for *Cx. quinquefasciatus*, there was a significantly higher number of larvae in pots containing a mixture of autoclaved soil and nonane compared to breeding-site soil (z = − 4.179, *P <* 0.0001).

### Influence of a mixture of nonane and soil from a natural breeding site

**Fig. 4:**
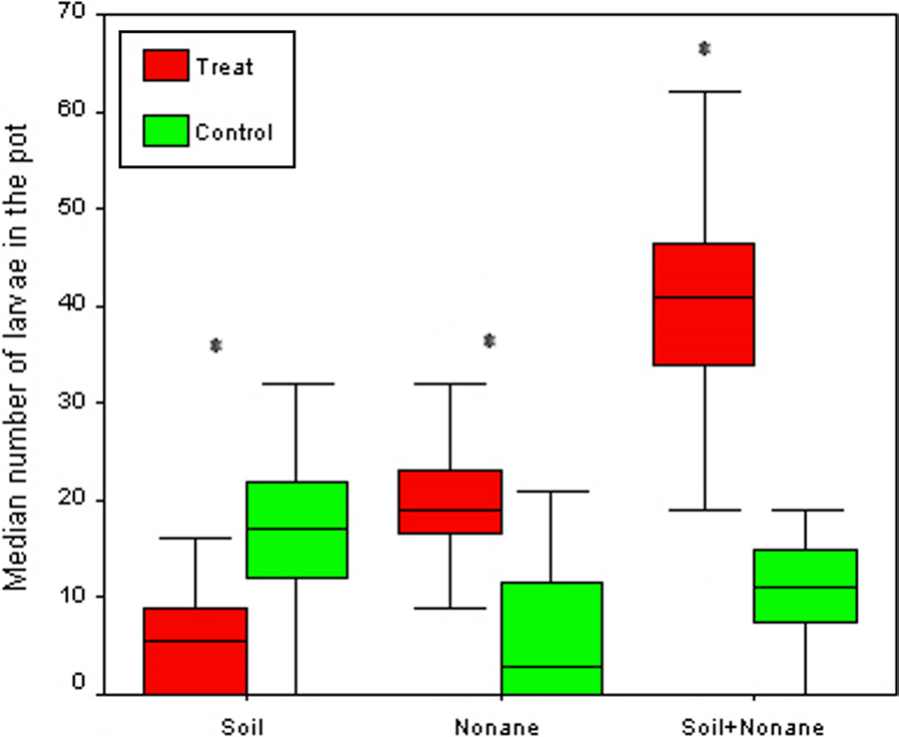
Oviposition response of *Culex quinquefasciatus* to a substrate containing soil, nonane or soil + nonane in a dual-choice field study. Distilled water + autoclaved soil was used as control. Boxplots represent the median number + quartiles of larvae per clay pot per day; Median and quartiles are given; asterisks indicate statistical differences between treatment and control for a given pair (*p < 0.05, Wilcoxon signed rank test)

In a choice test between distilled water + breeding-site soil + nonane against distilled water + breeding-site soil, a total of 1,248 *An. gambiae* larvae were found. Of these, 77.9% were found in pots containing distilled water + soil + nonane while 22.1% were found in pots containing distilled water + soil (Table 2; Fig. 4). Additionally, a total of 2,013 larvae of *Cx. quinquefasciatus* were found; 79.4% of these were found in pots containing distilled water + soil + nonane and 20.6% were found in pots containing distilled water + soil only (Table 3). The number of larvae found in pots containing distilled water with distilled water + soil + nonane was significantly higher than larvae found in distilled water + soil for both *An. gambiae* and *Cx. quinquefasciatus* (z = − 5.046, *P <* 0.0001 and z = − 5.512, *P <* 0.0001, respectively).

## Discussion

The oviposition pheromone nonane and emanations from breeding-site soil both attracted wild females of *An. gambiae* and *Cx. quinquefasciatus* to oviposit in the oviposition containers under field conditions. When both stimuli were present simultaneously, they acted synergistically. These results demonstrate the role that natural products play in the oviposition behaviour of wild populations of mosquitoes. In this study, *An. gambiae* selected clay pots above plastic or aluminium bowls for oviposition. It is likely that clay pots simulate the natural conditions that mosquitoes prefer for oviposition.

Similar to the present study, Herrera et al. [26], reported from a study in Kenya that oviposition substrates containing soil from a known breeding site produced significantly more larvae than a substrate without soil. This suggests that soil from a known breeding site contains and emits a chemical signal associated with microbial activity that attracts gravid mosquitoes and induces oviposition [49–53]. This is further supported by recent work from Eneh et al. [28], who demonstrated the emission of such cues from breeding-site soil and grasses. The authors suggest that these cues are derived from micro-organisms present in natural breeding sites and are likely to be inter-specific because they attracted both gravid *An. gambiae* s.l. and *Cx. quinquefasciatus*. In this study, autoclaved soil, in which all micro-organisms had been killed, did not induce oviposition behaviour of *An. gambiae* s.l. in contrast to untreated soil, to which the mosquitoes were attracted. This is consistent with other studies of mediation of oviposition-site selection by infochemicals of microbial origin [45, 54].

The effect of cues from micro-organisms present in breeding-site soil appears to be much stronger on *An. gambiae* s.l. than on *Cx. quinquefasciatus*, as the latter also laid eggs in pots containing autoclaved soil and even distilled water only, and thus behaved as a generalist species, in contrast to *An. gambiae* s.l.

The essay with nonane as a single cue (Fig. 3) indicated the mediation of oviposition behaviour of a natural population of *An. gambiae* s.l. by an intra-specific cue, as the volatile compound is produced by larvae and it attracts conspecific gravid mosquitoes to oviposit. The response elicited by nonane in this study is consistent with our previous findings on the effects of infochemicals emitted by early instars of *Anopheles coluzzii* on the oviposition behaviour of conspecific gravid females in the laboratory [55] and in the semi-field system with *An. gambiae* s.s. [40]. A similar response was observed with *Cx. quinquefasciatus* despite the fact that nonane originated from *An. gambiae*. This strongly suggests that the two species exploit the same chemical cues to locate suitable breeding sites. The presence of eggs or larvae of one species can thus act as an oviposition attractant for gravid mosquitoes of another species. It has previously been found that the oviposition pheromone of *Cx. quinquefasciatus* also attracted other culicine species, and this suggests that mosquitoes use a wide range of chemical cues in their oviposition behaviour [29, 39]. The finding that two different mosquito species, which are not genetically related, have evolved to respond to the same oviposition cues, is an interesting topic for future research, and suggests that olfactory receptors associated with oviposition are widely shared between different mosquito genera.

Mosquitoes use both inter- and intra-specific cues in locating suitable breeding sites. Previous studies on the role of the two cues reported conflicting findings. Some scholars thought that the intra-specific cue (pheromone) emitted by larvae from suitable breeding sites augments the attraction to inter-specific volatiles associated with microbial activity in natural anopheline pools [39, 54, 56]. Other authors reported that the presence of larvae in distilled water, even at low density, does not increase oviposition compared to distilled water without larvae [57, 58]. Similarly, in our previous studies [55], the presence of late-stage larvae, even at a low density, did not increase the oviposition response when compared to distilled water alone. However, it was observed that the presence of early instars increased oviposition compared to distilled water alone, suggesting that early instars emit intra-specific cues that are attractive to gravid mosquitoes. Nonane was identified from headspace volatiles collected from both early and late instars [40], and the present study showed that wild *An. gambiae* s.l. females prefer to oviposit in breeding sites emitting this compound.

Previous studies on the role of infochemicals emitted by larvae have cleared earlier doubts on whether the larval pheromone is stimulatory by itself (i.e., in the absence of the kairomone) or that the production of this pheromone occurs only in *An. gambiae* habitats containing suitable organic matter, microbes and algae [52, 54, 59]. Furthermore, in order to understand the interaction between an intra-specific signal (pheromone) and an inter-specific cue to the behaviour of *An. gambiae*, nonane and soil from a natural breeding site were combined in a choice assay and this combination was tested against soil or pheromone alone. The number of larvae that were found in water with nonane and breeding-site soil was higher than the number of larvae found in water with either nonane or soil alone. This indicates that inter- and intra-specific cues act synergistically when attracting gravid mosquitoes to lay eggs. Recently, cedrol was reported as an oviposition stimulant for *An. gambiae*, derived from breeding-site soil [39]. This compound was found to originate from grass present in the breeding site [26, 60]. Additionally, gravid *An. coluzzii* and *An. arabiensis* were found to be attracted to grass volatiles [61]. It is, therefore, possible that in the present study, plant- or soil-derived chemicals were responsible for the behavioural effect of the soil, of which cedrol to date is the only identified compound with proven activity in the field. The observed synergistic response on *An. gambiae* and *Cx. quinquefasciatus* may thus have been caused by the interaction of nonane and cedrol. The discovery of these chemical cues opens the way to the development of oviposition-based mediation/manipulation of populations of these harmful mosquito species.

The results of this study resemble the effect of the *Cx. quinquefasciatus* oviposition pheromone (5*R*,6*S*)-6-acetoxy-5-hexadecanolide and infochemicals derived from hay infusions, where a similar synergistic effect of both stimuli on gravid females was found [38]. The study suggests that there are several mechanisms ensuring that gravid females are guided effectively to sites that are suitable for egg laying and that the observed additive behavioural responses are a result of the perception of several interacting stimuli.

Being an infochemical of *An. gambiae* origin, nonane was expected to affect only gravid *An. gambiae* mosquitoes. In this study, significantly more culicine larvae were also found in pots containing nonane. This suggests that this compound can be used for the surveillance of other mosquito species as well. This finding is consistent with other studies, which suggest that *An. gambiae* and *Cx. quinquefasciatus* share breeding sites in many cases [62–66]. Further studies on the interaction of inter-and intra-specific cues of *An. gambiae* in the behaviour of other species will help to understand the universal role of infochemicals among various species of mosquitoes.

The findings from this study suggest that breeding-site derived infochemicals can be used for surveillance and control of mosquito vectors. In order for ovitraps to become effective as control agents in situations of multiple alternative oviposition sites (such as rice fields), the ovitrap should be at least as attractive, preferably more attractive than existing oviposition sites [67]. In the present study, nonane has been as attractive as breeding-site soil and the mixture of nonane and breeding-site soil induced a synergistic response. Therefore, if various signals are combined and emitted from specified breeding sites, gravid mosquitoes can be manipulated to lay their eggs on designated sites, which can easily be targeted for larvicide application. Therefore, the study reveals that the above attractants have the potential for use in developing a lure-and-kill system for the control of disease vectors.

## Conclusions

This study shows that nonane and emanations from natural breeding-site soil attract gravid females of *An. gambiae* s.l. and *Cx. quinquefasciatus* to sites containing these cues, and that both stimuli, once combined, act synergistically.

## Data Availability

Original data collected during the study are available with the authors upon request.
